# Commissioning measurements for photon beam data on three TrueBeam linear accelerators, and comparison with Trilogy and Clinac 2100 linear accelerators

**DOI:** 10.1120/jacmp.v14i1.4077

**Published:** 2013-01-07

**Authors:** Gloria P. Beyer

**Affiliations:** ^1^ Medical Physics Services LLC Tampa FL USA; ^2^ Medical Physics Services International Ltd. Cork Ireland

**Keywords:** linear accelerator, commissioning, photon beam data, standard beam dataset, flattening filter‐free data

## Abstract

This study presents the beam data measurement results from the commissioning of three TrueBeam linear accelerators. An additional evaluation of the measured beam data within the TrueBeam linear accelerators contrasted with two other linear accelerators from the same manufacturer (i.e., Clinac and Trilogy) was performed to identify and evaluate any differences in the beam characteristics between the machines and to evaluate the possibility of beam matching for standard photon energies. We performed a comparison of commissioned photon beam data for two standard photon energies (6 MV and 15 MV) and one flattening filter‐free (“FFF”) photon energy (10 FFF) between three different TrueBeam linear accelerators. An analysis of the beam data was then performed to evaluate the reproducibility of the results and the possibility of “beam matching” between the TrueBeam linear accelerators. Additionally, the data from the TrueBeam linear accelerator was compared with comparable data obtained from one Clinac and one Trilogy linear accelerator models produced by the same manufacturer to evaluate the possibility of “beam matching” between the TrueBeam linear accelerators and the previous models. The energies evaluated between the linear accelerator models are the 6 MV for low energy and the 15 MV for high energy. PDD and output factor data showed less than 1% variation and profile data showed variations within 1% or 2 mm between the three TrueBeam linear accelerators. PDD and profile data between the TrueBeam, the Clinac, and Trilogy linear accelerators were almost identical (less than 1% variation). Small variations were observed in the shape of the profile for 15 MV at shallow depths (< 5 cm) probably due to the differences in the flattening filter design. A difference in the penumbra shape was observed between the TrueBeam and the other linear accelerators; the TrueBeam data resulted in a slightly greater penumbra width. The diagonal scans demonstrated significant differences in the profile shapes at a distance greater than 20 cm from the central axis, and this was more notable for the 15 MV energy. Output factor differences were found primarily at the ends of the field size spectrum, with observed differences of less than 2% as compared to the other linear accelerators. The TrueBeam's output factor varied less as a function of field size than the output factors for the previous models; this was especially true for the 6 MV. Photon beam data were found to be reproducible between different TrueBeam linear accelerators well within the accepted clinical tolerance of ±2%. The results indicate reproducibility in the TrueBeam machine head construction and a potential for beam matching between these types of linear accelerators. Photon beam data (6 MV and 15 MV) from the Trilogy and Clinac 2100 showed several similarities and some small variations when compared to the same data measured on the TrueBeam linear accelerator. The differences found could affect small field data and also very large field sizes in beam matching considerations between the TrueBeam and previous linear accelerator models from the same manufacturer, but should be within the accepted clinical tolerance for standard field sizes and standard treatments.

PACS number: 87.56. bd

## I. INTRODUCTION

The TrueBeam is a new linear accelerator model manufactured by Varian. In this newest platform from Varian, many key elements differ significantly from those found in previous models. One of the key features is the availability of two types of photon beams: standard flattened filtered beams and flattening filter‐free (FFF) beams. The TrueBeam linear accelerator has a slightly different design for the head and related components from its predecessors. For example, the carrousel system has been modified to permit the use of several photon energies (flattened and FFF modes). This accelerator has an integrated bending magnet with an in‐air target instead of the vacuum‐sealed target found in the standard Varian models. The TrueBeam also contains a thicker primary collimator of slightly different design to permit sharper beam fall‐off, and uses an antibackscatter filter which can reduce the dose dependency on field size. This linear accelerator utilizes the same flattening filters for all standard photon energies as its predecessors, except for the 15 MV energy. The TrueBeam's 15 MV filter uses two different materials, while the Clinac and Trilogy models use a solid tungsten flattening filter.

The previous dual‐energy linear accelerator models from Varian include the Clinac models and the Trilogy model. The linear accelerator heads for these models are designed to the same specifications, but there are some implementation differences between the Clinac machines and the Trilogy model. The main difference is the availability in the Trilogy of a high‐dose rate (1000 MUs/min) 6 MV photon delivery mode, which has a separate small filter optimized for small field treatments. An important characteristic of the Clinac and Trilogy models has been the availability of “beam matching” when the linear accelerators were set within the manufacturer's specifications or set to a specific dataset within the manufacturer specification range.^(^ ^1^ ^)^ Such “beam‐matched” energies result in dosimetric characteristics between different linear accelerators that may properly be considered dosimetrically equivalent. The beam matching criteria are based on depth ionization curves, as well as profiles measured in a certain specified geometry. The vendor's product documentation describes the beam match concept and data analysis protocols in detail; they have also been the subject of previously published studies.^(^ ^2^ ^–^ ^4^ ^)^ One of the clear advantages of beam‐matching linear accelerators is the improved efficiency and flexibility in patient treatment for institutions with multiple linear accelerators. Beam‐match results and beam data reproducibility for Varian linear accelerators have previously been analyzed and presented.^(^ ^1^ ^–^ ^4^ ^)^

An additional characteristic from the reproducibility in the construction of the standard linear accelerator models is the availability of a beam dataset known as the Golden Beam Data (GBD). This reference dosimetric dataset is provided by the manufacturer (Varian Medical Systems). The accuracy of the Clinac dataset has previously been compared and evaluated.^(^ ^5^ ^)^ No reference dataset is currently available for the TrueBeam linear accelerator.

With the introduction of the new TrueBeam linear accelerator model, the additional FFF photon beam delivery mode will need to be considered in addition to the effects of the changes in the linear accelerator design. Several works have considered the beam characteristics and the benefits of using flattening filter‐free photon for radiation oncology treatments.^(^ ^6^ ^–^ ^9^ ^)^ Other works have explored the considerations of treatment planning for FFF modes.^(^ ^10^ ^–^ ^11^ ^)^ A recent work compared the data of regular photon beams with FFF beams for a Varian TrueBeam linear accelerator.^(^ ^12^ ^)^ However, no study has yet evaluated the beam characteristics of several TrueBeam linear accelerators for standard and FFF beam, or compared the dosimetric characteristics of previous linear accelerator models from the same manufacturer with the new TrueBeam linear accelerator.

This study evaluates the beam characteristics and the potential for beam‐matching capabilities of the TrueBeam linear accelerators. A comparison of two standard photon energies, 6 MV and 15 M V, and one flattening filter‐free (FFF) photon energy, 10 MV FFF (or 10 FFF) is performed for three different TrueBeam linear accelerators. The dosimetric and beam characteristics of two standard photon energies from the TrueBeam are then compared with the Clinac and Trilogy models from the same manufacturer and the possibility of “beam matching” between the TrueBeam and the standard Varian linear accelerator models is analyzed. The energies evaluated between the different linear accelerator models are the 6 MV for low energy and the 15 MV for high energy.

## II. MATERIALS AND METHODS

This study has been bifurcated for convenience. The first section compares the data from three separate TrueBeam linear accelerators. The second section compares the beam data measurements obtained from the TrueBeam linear accelerator with the data measurements from Trilogy and Clinac linear accelerators.

### A. TrueBeam data comparison

Beam data commissioning measurements of percent depth doses (PDDs), beam profiles, and output factors were performed on three Varian TrueBeam linear accelerators (Varian Medical Systems, Palo Alto, CA) located at three different locations. Measurements were performed for 6 MV and 15 MV standard photon energies and for 10 FFF photon energy. The linear accelerators were accepted following the manufacturer's recommended procedures, and each accelerator's mechanical parameters and beam data were confirmed to be within the manufacturer's specifications for normal operation. No attempt to match these machines was performed, as the data was acquired at different instances and the data comparison occurred after all relevant measurements had been obtained. Measurements for all three linear accelerators were performed using a CC13 $0.125\;{\text{cm}}^{3}$ ion chamber and IBA‐Wellhofer scanning phantom system (IBA Dosimetry, Barlett, TN). The chamber was offset to the effective point of measurement $\left(0.6*r_{\text{cav}}\right)$ for all photon beam data measurements performed.

An analysis of percent depth dose (PDD) data was performed to evaluate the energy match. The depth of dose maximum ($d_{\max}$) and PDD at 10 cm (${\text{PDD}}_{10}$) was evaluated for three field sizes: $4\times 4\;{\text{cm}}^{2}$, $10\times 10\;{\text{cm}}^{2}$, and $40\times 40\;{\text{cm}}^{2}$. An energy parameter value for comparison purposes was obtained by using a ${\text{TPR}}_{20/10}$ ratio. The TPR values were determined from the measured ${\text{PDD}}_{20\text{cm}}$ and ${\text{PDD}}_{10\text{cm}}$ data using an empirical approximation relation: ${\text{TPR}}_{20,10}=1.2661$ ${\text{PDD}}_{20,10}-0.0595$, where ${\text{PDD}}_{20,10}$ is the ratio of percent depth doses at 20 cm and 10 cm depths.^(^ ^13^ ^)^ An analysis of the mean and sample standard deviation was performed to evaluate the data variation. The 95% confidence interval (CI) on the mean was computed following the Student's t‐distribution.

An additional analysis was performed by comparing measurements for the crossplane beam profiles derived from two field sizes ($10\times 10\;{\text{cm}}^{2}$ and $40\times 40\;{\text{cm}}^{2}$) at two different depths (depth of approximately dose maximum and depth of 10 cm) and 100 cm SSD. Beam profile data analysis was performed by calculating the difference between the profile data for two linear accelerators (TrueBeam#2 and TrueBeam#3) to the remaining TrueBeam linear accelerator (TrueBeam#1). An analysis of the difference in beam profile value based on relative dose normalized to 100% at the central axis was performed. A further evaluation of the distance‐to‐agreement (DTA) was performed using a gamma analysis of the profiles.^(^ ^14^ ^)^ Profile data points were sampled at 1 mm spacing and the gamma analysis performed using criteria of dose difference (DD) of 2% and DTA of 1 mm.

Since previous data indicated variations in output factor data between energy‐matched linear accelerators,^(^ ^1^ ^)^ we evaluated additional output factor data. The total scatter factor data can determine variations in the beam filter construction and other characteristics of the linear accelerator head construction. We obtained relative output factor measurement data by using an isocentric setup at depth of 5 cm (95 cm SSD) for several field sizes ranging from $3\times 3\;{\text{cm}}^{2}$ to $40\times 40\;{\text{cm}}^{2}$. The resulting data were then averaged and compared to determine the variability between the different TrueBeam linear accelerators.

### B. Linear accelerator data comparison

For the second portion of our study, we measured PDDs, beam profiles, and output factors on two additional Varian linear accelerator models: a Trilogy and a Clinac 2100. Measurements were performed for 6 MV and 15 MV photon energies. These linear accelerators were fully accepted and determined to be operating within the manufacturer's specifications prior to the start of the beam data acquisition. No attempt to match these machines was performed, as the relevant data had been acquired at different times and at different facilities. Measurements for the two linear accelerators were performed using a CC13 $0.125\;{\text{cm}}^{3}$ ion chamber and IBA‐Wellhofer scanning phantom system. The chamber was offset to the effective point of measurement $\left(0.6*r_{\text{cav}}\right)$ for all beam data measurements performed.

The resulting dosimetric dataset from each linear accelerator was compared to the average data derived from the three TrueBeam linear accelerators. An analysis of percent depth dose (PDD) data was performed to evaluate the energy match. Data from three field sizes were analyzed: $4\times 4\;{\text{cm}}^{2}$, $10\times 10\;{\text{cm}}^{2}$, and $40\times 40\;{\text{cm}}^{2}$. The relative output factor measurement data were compared to the average data acquired from the TrueBeam linear accelerators to determine the variability between the different models.

The measured crossplane beam profiles were compared with the TrueBeam profiles. Beam profile penumbra (distance between the 80% and 20% relative dose points) and the field size definition (width at 50% relative dose) were then evaluated. Additional beam profile data analysis was performed by calculating the profile difference and gamma analysis (DD=2% and DTA=1 mm or 2 mm) with the TrueBeam profiles. The diagonal profile shape was compared to evaluate any additional effects from the differences in the collimator and head design.

## III. RESULTS & DISCUSSION

### A. TrueBeam data comparison

Measurements of PDDs between the three TrueBeam linear accelerators showed variability of less than 1.0% for the ${\text{PDD}}_{10}$ and variability within 2 mm for the $d_{\max}$ at the field sizes evaluated (Table 1). The statistical analysis presented in Table 1 is limited due to the small sample size. Specifically, the confidence intervals for the standard deviations values are necessarily broad. However, the reported 95% confidence interval of the mean can provide guidance for the expected reproducibility. The analysis of the TrueBeam TPR value showed minimum difference between the three different linear accelerators for the energies evaluated. It was expected that these parameters would be very similar since the PDD value at 10 cm for a $10\times 10\;{\text{cm}}^{2}$ field size is a key parameter in the beam quality specification during acceptance testing with a tolerance of 1%.

**Table 1:** TrueBeam energy match analysis.

*Energ (MV)*	*Data*	*Field Size (* cm×cm *)*	*TrueBeam #1*	*TrueBeam #2*	*TrueBeam #3*	*Average*	*St. Dev. (s)*	*95% CI Ave.*
		4×4	1.35	1.40	1.37	1.37	0.025	[1.31−1.44]
	$d_{\max}(\text{cm})$	10×10	1.47	1.40	1.36	1.41	0.056	[1.27−1.55]
		40×40	1.28	1.37	1.30	1.32	0.047	[1.20−1.43]
		4×4	61.3	61.5	61.4	61.4	0.100	[61.2−61.6]
6	${\text{PDD}}_{10}(\%)$	10×10	66.1	66.2	66.1	66.1	0.058	[66.0−66.3]
		40×40	71.7	71.9	71.9	71.8	0.115	[71.5−72.1]
		4×4	0.626	0.623	0.625	0.625	0.0015	[0.621−0.628]
	${\text{TPR}}_{20/10}$	10×10	0.666	0.667	0.665	0.666	0.0010	[0.664−0.668]
		40×40	0.746	0.744	0.745	0.745	0.0010	[0.743−0.747]
		4×4	2.78	2.90	2.80	2.83	0.064	[2.67−2.99]
	$d_{\max}$ (cm)	10×10	2.77	2.80	2.76	2.78	0.021	[2.72−2.83]
		40×40	2.07	2.00	2.00	2.02	0.040	[1.92−2.12]
		4×4	74.6	74.9	75.1	74.9	0.252	[74.2−75.5]
15	${\text{PDD}}_{10}(\%)$	10×10	76.7	76.8	76.6	76.7	0.100	[76.5−76.9]
	10	40×40	76.9	77.0	76.8	76.9	0.100	[76.7−77.1]
		4×4	0.731	0.732	0.729	0.731	0.0015	[0.727−0.734]
	${\text{TPR}}_{20/10}$	10×10	0.763	0.763	0.764	0.763	0.0006	[0.762−0.765]
		40×40	0.810	0.811	0.809	0.810	0.0010	[0.808−0.812]
		4×4	2.23	2.20	2.17	2.20	0.030	[2.13−2.27]
	$d_{\max}$ (cm)	10×10	2.26	2.25	2.13	2.21	0.072	[2.03−2.39]
		40×40	1.90	2.10	1.97	1.99	0.101	[1.74−2.24]
		4×4	67.5	67.9	67.5	67.6	0.231	[67.1−68.2]
10 FFF	${\text{PDD}}_{10}(\%)$	10×10	70.9	71.1	70.7	70.9	0.200	[70.4−71.4]
		40×40	73.1	73.2	73.2	73.2	0.058	[73.0−73.3]
		4×4	0.674	0.673	0.674	0.674	0.0006	[0.672−0.675]
	${\text{TPR}}_{20/10}$	10×10	0.705	0.703	0.707	0.705	0.0020	[0.700−0.710]
		40×40	0.744	0.741	0.745	0.743	0.0021	[0.738−0.749]

**Figure 1:**
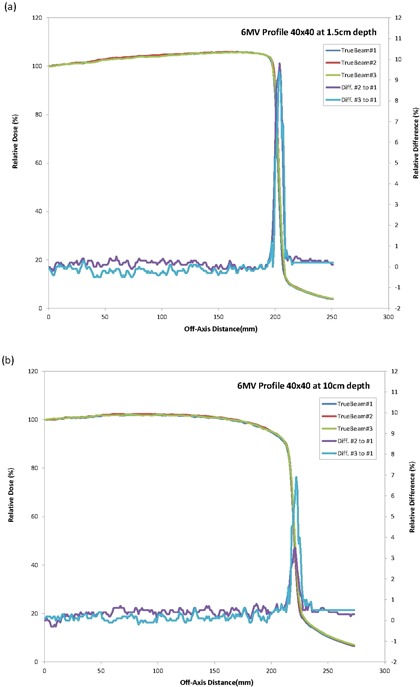
TrueBeam 6 MV crossprofiles and differences for $40\times 40\;{\text{cm}}^{2}$ field size at (a) 1.5 cm and (b) 10 cm.

**Figure 2:**
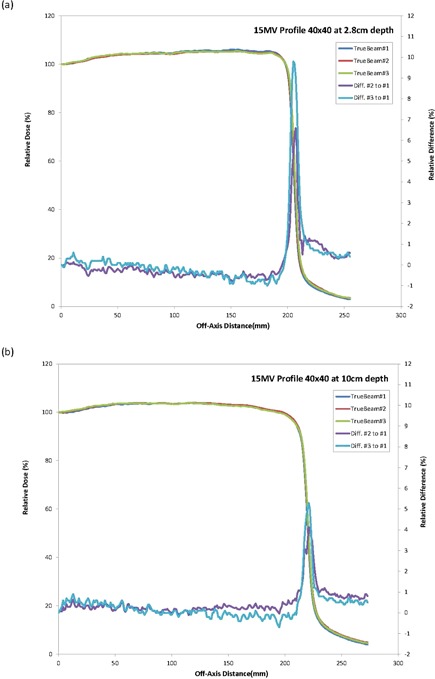
TrueBeam 15 MV crossprofiles and differences for $40\times 40\;{\text{cm}}^{2}$ field size at (a) 2.8 cm and (b) 10 cm.

**Figure 3:**
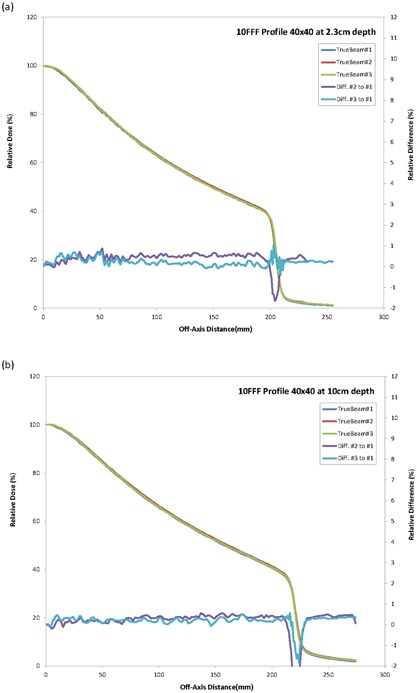
TrueBeam 10 FFF crossprofiles and differences for $40\times 40\;{\text{cm}}^{2}$ field size at (a) 2.3 cm and (b) 10 cm.

All profiles measured were also essentially identical for the three machines. The overlays of the profiles for the three machines were close to a single line (Figs. 1–3) indicating a similar beam quality and also high tolerance in the construction of the flattening filter for the standard photon energies. An analysis of the profile difference between TrueBeam#2 and #3 as compared with TrueBeam#1 showed variations <1.0% for areas of low gradient. The gamma analysis (DD=2% and DTA=1 mm) resulted in a 100% passing rate for the profiles evaluated.

The relative output factor in water at depth of 5 cm with respect to field size showed minimum variation (<0.5%) for all three TrueBeam linear accelerators (Table 2). The minimum variability of these measurements shows reproducibility in the collimator head construction of the TrueBeam linear accelerators.

**Table 2:** TrueBeam output factor data comparison.

*Energy*	*Field Size* (cm × cm)	*TrueBeam#1*	*TrueBeam Linear TrueBeam#2*	*Accelerator TrueBeam#3*	*Output Factors at 95 cm SAD Average*	*St. Dev. (s)*	*95% CI Ave.*
	3×3	0.877	0.875	0.878	0.877	0.0014	[0.873−0.880]
	4×4	0.904	0.903	0.904	0.904	0.0007	[0.902−0.905]
	6×6	0.946	0.945	0.946	0.946	0.0008	[0.944−0.948]
	8×8	0.977	0.977	0.978	0.977	0.0003	[0.976−0.978]
6 MV	10×10	1.000	1.000	1.000	1.000	0.0000	[1.000−1.000]
	15×15	1.040	1.038	1.040	1.039	0.0010	[1.037−1.042]
	20×20	1.067	1.066	1.067	1.067	0.0005	[1.065−1.068]
	30×30	1.102	1.101	1.103	1.102	0.0011	[1.100−1.105]
	40×40	1.115	1.114	1.117	1.115	0.0015	[1.112−1.120]
	3×3	0.874	0.873	0.876	0.874	0.0019	[0.870−0.879]
	4×4	0.913	0.913	0.915	0.914	0.0008	[0.912−0.916]
	6×6	0.955	0.954	0.955	0.955	0.0007	[0.953−0.957]
	8×8	0.981	0.981	0.981	0.981	0.0003	[0.980−0.982]
15 MV	10×10	1.000	1.000	1.000	1.000	0.0000	[1.000−1.000]
	15×15	1.030	1.029	1.030	1.030	0.0005	[1.028−1.031]
	20×20	1.050	1.049	1.049	1.049	0.0004	[1.048−1.050]
	30×30	1.075	1.075	1.075	1.075	0.0003	[1.074−1.075]
	40×40	1.083	1.085	1.082	1.083	0.0015	[1.079−1.087]
	3×3	0.921	0.919	0.922	0.920	0.0015	[0.917−0.924]
	4×4	0.947	0.946	0.948	0.947	0.0008	[0.945−0.949]
	6×6	0.973	0.973	0.974	0.974	0.0007	[0.972−0.975]
	8×8	0.990	0.989	0.989	0.989	0.0006	[0.988−0.991]
10 FFF	10×10	1.000	1.000	1.000	1.000	0.0000	[1.000−1.000]
	15×15	1.019	1.018	1.018	1.018	0.0007	[1.016−1.020]
	20×20	1.030	1.030	1.030	1.030	0.0004	[1.029−1.031]
	30×30	1.042	1.043	1.044	1.043	0.0011	[1.040−1.046]
	40×40	1.047	1.047	1.048	1.047	0.0002	[1.047−1.048]

Similar results in photon beam data reproducibility as obtained in this study had been reported for the previous Clinac linear accelerator model. A study showed photon beam data measurements within 2% variability, with most beam parameters analyzed within 1% variability.^(^ ^2^ ^)^ It should be noted that since no attempt on “beam matching” these TrueBeam linear accelerators was performed, the dataset could probably be fine‐tuned to a greater agreement, if necessary, as has been performed for previous linear accelerator models.^(^ ^1^ ^)^

### B. Linear accelerator data comparison

Measurements of PDD showed relative variability less than 1.0% for the ${\text{PDD}}_{10}$ and within 2 mm for the $d_{\max}$ between the average TrueBeam, on the one hand, and the Clinac 2100 and the Trilogy, on the other hand, at the field sizes evaluated (Table 3). The TPR data showed minimum difference between the linear accelerators. The TPR data for the $10\times 10\;{\text{cm}}^{2}$ field size was found to be well within the TrueBeam data confidence interval, indicating no significant energy difference between the linear accelerators for the energies evaluated. It was expected that these parameters would be very similar since the PDD value and tolerance at 10 cm for a $10\times 10\;{\text{cm}}^{2}$ field size as specified in the acceptance documents from the manufacturer are the same for each of the linear accelerators tested.

**Table 3:** Energy match analysis between TrueBeam, Clinac, and Trilogy linear accelerators.

*Energy (MV)*	*Data*	*Field Size* (cm × cm)	*TrueBeam*	*Clinac*	*Trilogy*
		4×4	1.37	1.38	1.41
	$d_{\max}$ (cm)	10×10	1.41	1.41	1.44
		40×40	1.32	1.20	1.29
		4×4	61.4	61.4	61.7
6	${\text{PDD}}_{10}$ (%)	10×10	66.1	66.2	66.1
		40×40	71.8	71.3	71.5
		4×4	0.625	0.627	0.626
	${\text{TPR}}_{20/10}$	10×10	0.666	0.667	0.668
		40×40	0.745	0.741	0.746
		4×4	2.83	3.00	2.80
	$d_{\max}$ (cm)	10×10	2.78	2.60	2.59
		40×40	2.02	2.15	2.08
		4×4	74.9	75.1	74.8
15	${\text{PDD}}_{10}$ (%)	10×10	76.7	76.6	76.5
		40×40	76.9	77.3	77.5
		4×4	0.731	0.731	0.733
	${\text{TPR}}_{20/10}$	10×10	0.763	0.762	0.763
		40×40	0.810	0.807	0.811

**Figure 4:**
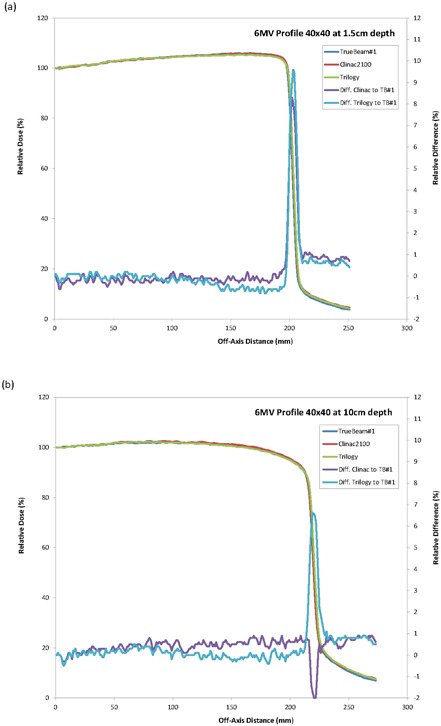
Crossprofiles and differences for a $40\times 40\;{\text{cm}}^{2}$ field size for 6 MV at depth of (a) 1.5 cm and (b) 10 cm.

**Figure 5:**
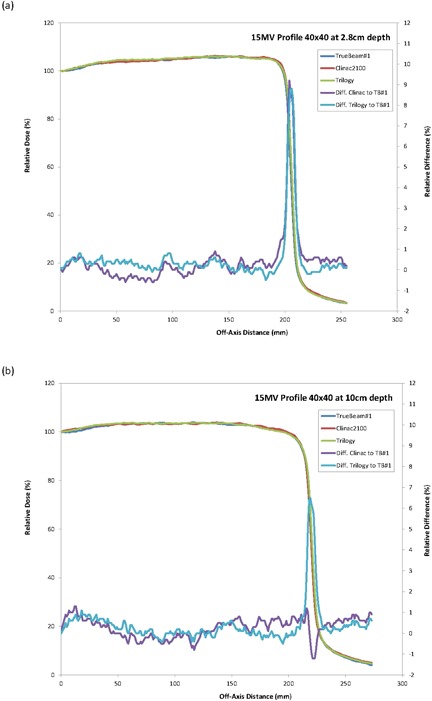
Crossprofiles and differences for a $40\times 40\;{\text{cm}}^{2}$ field size for 15 MV at depth of (a) 2.8 cm and (b) 10 cm.

The gamma analysis of the profiles (DD=2% and DTA=1 mm) resulted in a passing rate greater than 99.0% for all cases except at depth of 2.8 cm for 15 M V, where the passing rate was greater than 98.0%. To further evaluate the profile differences, the profile data for one TrueBeam was graphically superimposed to the Clinac 2100 and Trilogy profiles (Figs. 4 and 5). Some variations were observed in the shape of the profile for 15 MV within the field and were more noticeable at the depth close to dose maximum. This can be clearly observed on the dose difference plots. This is probably caused by a difference in the flattening filter design for the 15 MV. However, even with the change in the flattening filter, the square field crossprofiles from the TrueBeam linear accelerators matched closely with the standard Varian linear accelerators; the gamma analysis with DD=2% and DTA=2 mm resulted in a 100.0% passing rate.

**Figure 6:**
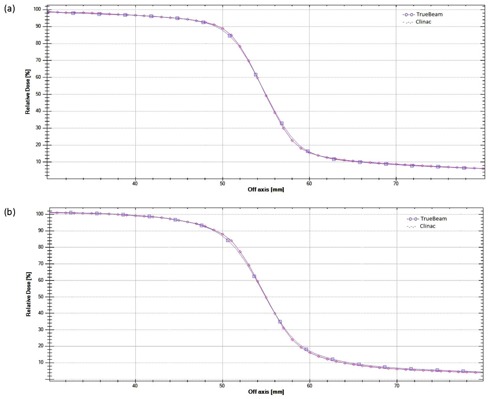
Penumbra profiles for $10\times 10\;{\text{cm}}^{2}$ for a TrueBeam and Clinac at 10 cm depth for (a) 6 MV and (b) 15 MV.

A difference in the penumbra shape was observed between the TrueBeam and the other linear accelerators, with the TrueBeam data resulting in a slightly larger penumbra width (Fig. 6). The penumbra for the Clinac and the Trilogy were slightly sharper than that for the TrueBeams for most cases (Table 4). It should be noted that no attempt was made to match the jaw positioning calibration between the different machines. However, the scanned field width variations were within 2 mm for the profiles evaluated.

**Table 4:** Beam profile analysis for TrueBeam, Clinac, and Trilogy linear accelerators.

Energy=6 MV		10 cm×10 cm *Field Size*		40 cm×40 mm *Field Size*	
*Depth (cm)*	*Linear Accelerator*	*Penumbra (mm)*	*Field Width (cm)*	*Penumbra (mm)*	*Field Width (cm)*
	TrueBeam#1	5.7	10.17	6.0	40.77
	TrueBeam#2	5.6	10.18	5.9	40.78
1.5	TrueBeam#3	5.5	10.17	5.8	40.79
	Clinac 2100	5.4	10.15	5.7	40.72
	Trilogy	5.5	10.16	5.7	40.91
	TrueBeam#1	7.2	11.03	10.1	44.14
	TrueBeam#2	7.0	11.03	10.0	44.11
10	TrueBeam#3	7.0	11.05	9.9	44.19
	Clinac 2100	6.8	11.02	10.0	44.16
	Trilogy	6.8	11.03	10.1	44.29

Energy=15 MV		10 cm×10 cm *Field Size*		40 cm×40 mm *Field Size*	
*Depth (cm)*	*Linear Accelerator*	*Penumbra (mm)*	*Field Width (cm)*	*Penumbra (mm)*	*Field Width (cm)*
	TrueBeam#1	6.8	10.34	6.9	41.19
	TrueBeam#2	6.8	10.34	7.3	41.13
2.8	TrueBeam#3	6.7	10.33	7.0	41.14
	Clinac 2100	6.4	10.36	6.7	41.25
	Trilogy	6.4	10.32	6.9	41.26
	TrueBeam#1	7.8	11.06	8.9	44.08
	TrueBeam#2	7.7	11.05	9.2	44.07
10	TrueBeam#3	7.6	11.07	8.8	44.19
	Clinac 2100	7.5	11.08	8.6	44.26
	Trilogy	7.3	11.04	8.8	44.21

Since the TrueBeam, Trilogy, and Clinac 2100 linear accelerators all use the same collimator jaw design and materials, the slightly wider penumbra on the TrueBeam profiles is probably caused by the different design and materials of the linear accelerator head affecting the beam scattering and by the different design of the bending magnet affecting the electron spot size at the X‐ray target (Personal communication, Varian Medical Systems, July 20, 2012). Additional small field data measurements and comparisons are needed to determine the possible effects of the penumbra difference for small field treatments. Similarly, additional validations using beam modeled data are necessary to determine the possible implications for treatment planning.

**Figure 7:**
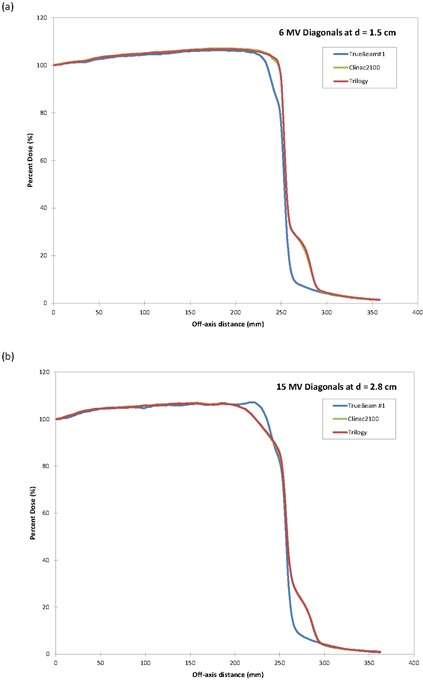
Diagonal profiles for a $40\times 40\;{\text{cm}}^{2}$ field size for (a) 6 MV at 1.5 cm and (b) 15 MV at 2.8 cm depth.

The diagonal scans demonstrated a significant difference in the profile shape at a distance greater than 20 cm from the central axis which was most notable for the 15 MV photon energy (Fig. 7). The additional “peak” in the shape of the 15 MV diagonal profile for the TrueBeam was probably caused by the difference in the shape of the flattening filter. The other differences observed outside the field for both photon energies are probably caused by the thicker primary collimator in the TrueBeam as compared with both the Trilogy and the Clinac models. The TrueBeam primary collimator was designed to give a sharper field drop‐off which was clearly observed in the diagonal profiles of the TrueBeam as compared to the other standard linear accelerators.

**Figure 8:**
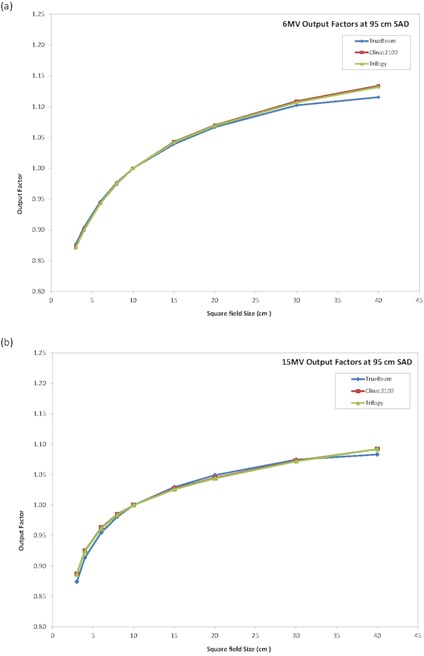
Square field output factors at 95 cm SAD for (a) 6 MV and (b) 15 M V.

The analysis of the output factors from the TrueBeam average data showed some differences greater than 1% but less than 2% for smaller and larger field sizes when compared to the Clinac and the Trilogy linear accelerators. This can clearly be seen in the graphical representation of the output factors as a function of field size (Fig. 8). It was noted that the TrueBeam output factor values varied less as a function of field size, especially for the 6 MV beam, than that of the Clinac or the Trilogy linear accelerators. The difference in the field size dependence of the output factors is probably related to the antibackscatter filter introduced in the TrueBeam to reduce the dose dependency on field size. Other differences in the head construction of the TrueBeam could also have caused the differences in the output factors when compared to the Clinac 2100 and Trilogy linear accelerators.

In summary, it can be noted that the Clinac 2100 and Trilogy photon beam data have some differences with the data from the new TrueBeam linear accelerator, but also several similarities. With the exception of the diagonal profiles, the photon beam data variation was less than 2%. The differences encountered are mostly related with the changes in the head design of the new linear accelerator model. These differences could possibly affect small field data and also very large field sizes in beam‐matching considerations, but should be within the accepted clinical tolerance for standard field sizes and standard treatments.

## IV. CONCLUSIONS

Photon beam data were found to be reproducible between different TrueBeam linear accelerators indicating reproducibility in the filter and machine head construction. The consistency of the beam data implies that a single beam dataset could be established for a set of TrueBeam linear accelerators within a clinic, indicating the potential for beam matching between such machines in the clinical environment.

Photon beam data PDDs from TrueBeam (6 and 15 MV) as compared with the Trilogy and Clinac 2100 were very similar. The profiles from the TrueBeam (6 and 15 MV) showed some small differences as compared with the Trilogy and Clinac 2100. Some difference in shape of the profile within the field was observed for 15 MV. The TrueBeam profiles evaluated showed a slightly wider penumbra. Differences were also found in the shape of the diagonal profiles at distances greater than 20 cm from the central axis. Some differences (<2%) were found in the output factors, mainly for the small and large field sizes, with the TrueBeam output factor data varying less as a function of field size.

These results could affect small field data and also very large field sizes in beam‐matching considerations between the TrueBeam and previous linear accelerator models from the same manufacturer. Additional studies involving the equivalence of treatment planning modeling with beam data from each linear accelerator type are necessary to determine the range of clinical significance for beam‐matching considerations.

## ACKNOWLEDGMENTS

The author would like to acknowledge the following people for their assistance in making this work possible: Jan Pursley and Rachel E. Younger (Medical Physics Services, LLC); Rick Dunia (Fresno Cancer Center); Y. Ray Wang (Nevada Cancer Institute); Francesca Attanasi (Casa di Cura San Rosore); Sandra Viera and Dalila Mateus (Champalimaud Foundation); Peter McBride (St. Lukes Radiation Oncology); Radka Stoyanova (University of Miami); and finally Andrew Bebb (Education Department) and Michelle Svatos (Translational Research) at Varian Medical Systems.
